# Prediction of microalbuminuria from proteinuria in chronic kidney disease due to non-diabetic lifestyle-related diseases: comparison with diabetes

**DOI:** 10.1007/s10157-021-02027-6

**Published:** 2021-03-03

**Authors:** Makoto Ogi, Takuya Seto, Yoshinori Wakabayashi

**Affiliations:** Department of Internal Medicine, Yuurinkouseikai Fuji Hospital, 1784 Niihashi, Gotemba, Shizuoka 412-0043 Japan

**Keywords:** Lifestyle-related disease, Chronic kidney disease, Non-diabetes mellitus, Microalbuminuria, Proteinuria

## Abstract

**Background:**

To suppress increases in kidney failure and cardiovascular disease due to lifestyle-related diseases other than diabetes, early intervention is desirable. We examined whether microalbuminuria could be predicted from proteinuria.

**Methods:**

The participants consisted of adults who exhibited a urinary protein-to-creatinine ratio (uPCR) of < 0.5 g/gCr and an eGFR of ≥ 15 ml/min/1.73 m^2^ in their spot urine at their first examination for lifestyle-related disease. Urine was tested three times for each case, with microalbuminuria defined as a urinary albumin-to-creatinine ratio (uACR) of 30–299 mg/gCr, at least twice on three measurements. Youden’s Index was used as an index of the cut-off value (CO) according to the ROC curve.

**Results:**

A single uPCR was useful for differentiating normoalbuminuria and micro- and macroalbuminuria in patients with non-diabetic lifestyle-related diseases. Regarding the GFR categories, the CO of the second uPCR was 0.09 g/gCr (AUC 0.89, sensitivity 0.76, specificity 0.89) in G1-4 (*n* = 197) and 0.07 g/gCr (AUC 0.92, sensitivity 0.85, specificity 0.88) in G1-3a (*n* = 125). Using the sum of two or three uPCR measurements was more useful than a single uPCR for differentiating microalbuminuria in non-diabetic lifestyle disease [CO, 0.16 g/gCr (AUC 0.91, sensitivity 0.85, specificity 0.87) and 0.23 g/gCr (AUC 0.92, sensitivity 0.88, specificity 0.84), respectively].

**Conclusion:**

Microalbuminuria in Japanese individuals with non-diabetic lifestyle-related diseases can be predicted from the uPCR, wherein the CO of the uPCR that differentiates normoalbuminuria and micro- and macroalbuminuria was 0.07 g/gCr for G1-3a, while that in G3b-4 was 0.09 g/gCr.

## Introduction

A GFR of < 60 ml/min/1.73m^2^ or a urinary albumin-to-creatinine ratio (uACR) of ≥ 30 mg/gCr is independent risk factor for all-cause mortality, cardiovascular mortality, kidney failure, acute kidney injury, and kidney disease progression in chronic kidney disease (CKD) [1, 2]. In Japan, diabetic nephropathy is the most common primary disease among the prevalent dialysis patients, and the rate of nephrosclerosis due to lifestyle-related diseases other than diabetes has been increasing [3], making it necessary to prevent kidney failure by early intervention for microalbuminuria, as albuminuria is a risk factor for CKD progression and kidney failure [1, 2, 4]. Microalbuminuria is also a risk factor for cardiovascular diseases and death in lifestyle-related diseases [5–7], such as diabetes [4], hypertension [8], and metabolic syndrome [9], as well as in the general population [10]. Furthermore, albuminuria is a risk factor for the development of Alzheimer’s disease and vascular dementia [11], which are common reasons for individuals requiring care in an aging society.

CKD is defined as an abnormal kidney structure and function, which persist for more than 3 months in KDIGO [1]. One marker of kidney damage is albuminuria ≥ 30 mg/24 h or ACR ≥ 30 mg/gCr [1]. CKD is classified based on cause, GFR, and albuminuria category, and the boundary between A1 (normal to mildly increased) and A2 (moderately increased) is albuminuria 30 mg/24 h or uACR 30 mg/gCr for all primary diseases [1]. Proteinuria, with a urinary protein-to-creatinine ratio (uPCR) of 150–500 mg/gCr is considered to be equivalent to A2 albuminuria (30–300 mg/gCr) [1]. On the other hand, in CKD staging in Japan, when the primary disease is diabetes, the boundary between A1 and A2 is a uACR of 30 mg/gCr; however, in CKD due to non-diabetic lifestyle-related diseases, the boundary between A1 and A2 is a uPCR of 0.15 g/gCr [2].

While dipstick proteinuria is a risk factor for kidney failure [12], along with cardiovascular disease and death, albuminuria is a superior predictor of cardiovascular disease [13]; thus, a method for predicting microalbuminuria is desired. However, the dipstick method cannot adequately evaluate small amounts of albuminuria [14]. Although there have been reports on the relationship between proteinuria and albuminuria [14–20], many reports indicate that it is difficult or inaccurate to predict microalbuminuria based on proteinuria [14, 17, 19]. It has been reported that the uPCR and uACR show a similar association with the risk of kidney disease progression in children with CKD without diabetes [20]. However, there appear to be no sufficient studies on whether a uPCR of 0.15 g/gCr is optimal as a boundary value for detecting patients with a uACR of ≥ 30 mg/gCr among adults with non-diabetic lifestyle-related diseases in Japan.

The objective of this study was to investigate whether proteinuria could predict albuminuria of < 300 mg/gCr, including a uACR of 30 mg/gCr, which is the boundary value for A1 and 2 in non-diabetic lifestyle-related diseases. We also aimed to compare the relationship to that in patients with diabetes.

## Methods

The subjects included Japanese patients of ≥ 18 years of age who visited the Yuurinkousekusei Fuji Hospital during the period from October 2017 to September 2019 for lifestyle-related diseases or CKD due to the diseases or aging, with an eGFR of ≥ 15 ml/min/1.73 m^2^ and a uPCR of < 0.5 g/gCr at initiation. Patients with urinary tract infection, nephritis, hereditary renal disease, paraproteinemia, or cancer were excluded. Patients who gave their written consent to participate in the study were chosen as subjects. A history of lifestyle-related diseases and smoking was noted and their blood pressure, BMI, waist circumference, blood glucose, HbA1c, serum uric acid, LDL-C, triglycerides, and non-HDL cholesterol levels were measured or the results of a health checkup were noted. Proteinuria and urinary occult blood were detected using the dipstick method, and urinary sediment was performed. At the same time, urinary protein and creatinine were measured to calculate uPCR. eGFR was calculated from age, sex, and serum creatinine [2].

Albumin was measured in the same urine sample of patients with a uPCR of < 0.5 g/gCr to determine the uACR. Proteinuria and albuminuria were measured three times on different days, including the first measurement, with the second and third measurements performed using early morning spot urine [1]. Patients whose proteinuria increased to ≥ 0.5 g/gCr on the second or third measurement were not excluded. The patients visited the clinic up to once every 3 months, depending on their CKD stage, with the eGFR calculated on each visit. Patients whose eGFR decreased by ≥ 30% within 3 months were excluded.

Microalbuminuria was defined as a uACR of 30–299 mg/gCr on at least two of three measurements, while normoalbuminuria was defined as a uACR of < 30 mg/gCr on at least two of three measurements, and macroalbuminuria was defined as a uACR of ≥ 300 mg/gCr on at least two of three measurements. When uACR was < 30 mg/gCr, 30–299 mg/gCr, and ≥ 300 mg/gCr, once each, then this condition was defined as microalbuminuria in this study. The average eGFR during the period was used to determine the GFR category in each case.

Urinary protein was measured using the pyrogallol red method (AR WAKO microTP-AR, Wako Pure Chemical Industries), urinary albumin was measured using an immunoturbimetric method (AutoWako Microalbumin, Wako Pure Chemical Industries), and serum and urine creatinine were measured using the enzyme method (L type WAKO CREM, FUJIFILM Wako Pure Chemical Corporation) with an autoanalyzer (TBA-2000FR).

Student's *t *test and the chi-squared test were used to compare clinical symptoms, and the Mann–Whitney U test, and Kruskal–Wallis and Steel–Dwass tests were used to compare the urinary protein, urinary albumin, urinary creatinine, uACR, and uPCR values for each category. The median uACR and IQR ranging from uPCR 0.01 g/gCr to 0.16 g/gCr were examined for each uPCR 0.01 g/gCr in all cases, non-diabetics and diabetics, respectively. The distributions of uACR and uPCR were found to be highly skewed (skewness test for normality, p < 0.0001); thus, the uACR and uPCR were log-transformed. The relationship between log uPCR and log median uACR was analyzed by a linear spline. The change in slope within the range between knots was investigated. We also analyzed the nonlinear association using a restricted cubic spline. Akaike’s information criterion (AIC) was used for the data-based choice of the number of knots, and the knots were placed at standard locations [21].

The receiver operating characteristic (ROC) curve was used to determine whether a uACR of ≥ 30 mg/gCr and microalbuminuria could be differentiated by a single uPCR or a sum of two or three uPCRs and a single uACR, to obtain the cut-off value (CO), sensitivity (Sn), and specificity (Sp), with the largest Youden’s index (YI) value, using the area under the curve (AUC). If the YI was the same, the CO with the larger Sn was adopted. The correlation between changes in the uACR and uPCR was examined by a simple regression analysis.

The subjects for the examination on the intra- and interday variance of uPCR and uACR of the same sample include patients who were regularly visiting a hospital with lifestyle-related disease in December 2020, had a uPCR of less than 0.5 g/gCr at their last outpatient visit within 3 months prior, and used the morning outpatient service. In the morning of day 0, serum creatinine was measured and a urinalysis and sediment testing of the morning urine was carried out. The first measurement was performed in the morning of day 0 and urinary protein, urinary albumin, and urinary creatinine were measured at the same time. The specimens were then stored in the refrigerator at 5 to 7 degrees. The second measurement was carried out 3 h after but within 4 h of the first measurement on the same day, with the urinary protein, albumin, and creatinine measured at the same time after leaving the specimens for 15 min at room temperature. Thereafter, we plugged the specimens, wrapped them with parafilm, and cryopreserved them at -80 degrees. The third and fourth measurements were carried out on day 3 or 4. The specimens were thawed with running water at 11:30 AM and left at room temperature from 11:40 AM, after which the urinary protein, albumin, and creatinine were measured at 12:00 PM, twice consecutively within 3 min. Patients with uPCR ≥ 0.5 g/gCr in the morning of day 0 were not excluded.

For urinary protein, urinary albumin, urinary creatinine, uPCR, and uACR, we calculated the mean of the two measurements used for comparison. For urinary protein, urinary albumin, and urinary creatinine, the mean values were classified into three groups in ascending order. For uPCR and uACR, the mean values of uPCR were divided into two groups: less than 0.15 g/gCr; and 0.15 g/gCr or more. We then calculated the intraday correlation and interday correlation within each group, the median, and the IQR of the bias of the later measurements against the previous measurements. In each of the four measurements, we calculated the CO of uPCR and urinary protein concentration estimating a uACR of 30 mg/gCr or more, which were measured simultaneously. The CO of uPCR estimating the mean of the four uACR values ≥ 30 mg/gCr was also calculated and used for comparison.

All statistical analyses were performed using the BellCurve for Excel (Social Survey Research Information Co., Ltd., Tokyo, Japan), SigmaStat Statistics (Systat software, Inc., USA) and Stata MP version16 (StataCorp LP, College Station, Texas).

## Results

The subjects included 197 patients with non-diabetic lifestyle-related diseases (age, 68.9 ± 14.8 years) and 106 patients with diabetes (age, 67.1 ± 11.6 years). Table 1 shows the primary lifestyle-related diseases, GFR, and albuminuria category.

**Table 1:** Characteristics of patients

	Non-diabetes mellitus		Diabetes mellitus		*p* value
	(*n* = 197)	(%)	(*n* = 106)	(%)	
Age (years)	68.9 ± 14.8		67.1 ± 11.6		0.85
Sex (m:f)	124:73		70:36		0.59
Obesity	61	(31.0)	46	(43.4)	0.031
Waist circumference					
Male ≥ 85 cm	72	(58.1)	49	(70.0)	0.10
Female ≥ 90 cm	22	(30.1)	16	(44.4)	0.14
Smoking					
Previous	59	(29.9)	35	(33.0)	0.58
Current	22	(11.2)	13	(12.3)	0.78
Complicated disease					
Hypertension	126	(64.0)	82	(77.4)	0.017
Dyslipidemia	113	(57.4)	64	(60.4)	0.61
Hyperuricemia	86	(43.7)	23	(21.7)	0.00015
Dipstick proteinuria					
(−)	143	(72.6)	81	(76.4)	0.47
(±)	30	(15.2)	13	(12.3)	0.48
1( +)	21	(10.7)	11	(10.4)	0.94
2( +)	3	(1.5)	1	(0.9)	0.67
Gfr category					
G1	5	(2.5)	12	(11.3)	0.0015
G2	47	(23.9)	45	(42.5)	0.00079
G3a	73	(37.1)	26	(24.5)	0.026
G3b	44	(22.3)	16	(15.1)	0.13
G4	28	(14.2)	7	(6.6)	0.048
Albuminuria category					
Normoalbuminuria	93	(47.2)	51	(48.1)	0.88
Microalbuminuria	98	(49.7)	55	(51.9)	0.72
Macroalbuminuria	6	(3.0)	0		0.07
Time between mesurements (days)					
1–2	84.4 ± 60.8		75.1 ± 56.5		0.97
2–3	75.0 ± 61.8		66.2 ± 45.8		0.79

Microalbuminuria: urinary albumin-to-creatinine ratio 30–299 mg/gCr at least twice for 3 measurements. Data are shown as the mean ± standard deviation

*CKD* chronic kidney disease; *GFR* glomerular filtration rate

Table 2 shows the uACR and uPCR for each GFR category. While proteinuria was less than the measured sensitivity 44 times (7.4%) among non-diabetics and 18 times (5.7%) among diabetics, albuminuria was less than the measured sensitivity 1 time (0.7%) among non-diabetics. The uACR values did not differ according to the GFR category, and the uPCR values in G4 were higher in comparison to those in G1 among patients with non-diabetic lifestyle-related diseases. In diabetics, the uACR and uPCR values in G3b and 4 were higher in comparison to those in G1–3a. When uPCR/uACR was assessed, with the exception of one case in which uACR was below the level of sensitivity, the uPCR/uACR values were lower in non-diabetics than in diabetics in G1 and higher in G4(Table 2).

**Table 2:** uACR, uPCR and uPCR/uACR in patients with and without diabetes mellitus according to GFR category

GFR category	n	uACR (mg/gCr)	uACR undetected	uPCR (g/gCr)	uPCR undetected	uPCR/uACR (g/gCr)/(g/gCr)
Non-diabetes mellitus						
G1	15	48.2 ± 29.8*	0	0.076 ± 0.057	3(20.0)	1.53 ± 0.94*
G2	141	71.6 ± 95.8	1(0.7)	0.112 ± 0.124	9(6.4)	2.86 ± 3.76^d^
G3a	219	55.8 ± 75.9	0	0.102 ± 0.117	19(8.3)	2.91 ± 3.12
G3b	132	74.4 ± 82.4	0	0.120 ± 0.116*	8(6.1)	2.51 ± 2.47
G4	84	77.0 ± 114.3**	0	0.150 ± 0.198c1*	5(6.0)	3.14 ± 3.15*

GFR category	n	uACR (mg/gCr)	uACR undetected	uPCR (mg/gCr)	uPCR undetected	uPCR/uACR (g/gCr)/(g/gCr)
Diabetes mellitus						
G1	36	31.6 ± 28.6	0	0.072 ± 0.075	2(5.6)	3.04 ± 2.60
G2	135	37.4 ± 35.3	0	0.074 ± 0.064	11(8.1)	2.67 ± 2.05
G3a	78	52.7 ± 62.8	0	0.103 ± 0.105	5(5.7)	2.56 ± 2.04
G3b	48	87.6 ± 83.4^abc1^	0	0.168 ± 0.142^abc1^	0	2.44 ± 1.56
G4	21	125.5 ± 97.3^abc2^	0	0.180 ± 0.119^abc2^	0	1.91 ± 1.12

Data are shown as the mean ± standard deviation. Undetected uACR and uPCR were regarded as 0 mg/gCr and 0/gCr, respectively

*uACR* urinary albumin-to-creatinine ratio; uPCR, urinary protein-to-creatinine ratio; GFR, glomerular filtration rate

*p < 0.05, **p < 0.01 vs. diabetic patients by the Mann–Whitney's U test

^a^*p* < 0.01 vs. G1

^b^*p* < 0.01 vs. G2

^c1^*p* < 0.05 vs. C3a

^c2^*p* < 0.01 vs. C3a by the Steel–Dwass test

^d^One patient with undetected albuminuria was excluded

### Relationship between the uPCR and uACR

The study on the relationship between the uPCR and the median uACR indicated that the median uACR increased as the uPCR increased in all patients, non-diabetic patients and diabetic patients, with the median uACR exceeding 30 mg/gCr at uPCR 0.09 g/gCr in all patients and non-diabetic patients and at uPCR 0.07 g/gCr in diabetic patients. The median uACR corresponding to uPCR 0.15 g/gCr was 71 mg/gCr in the overall patients, 72.5 mg/gCr in non-diabetic patients, and 69.0 mg/gCr in diabetic patients (Table 3).

**Table 3:** Association of uPCR (g/gCr), the median uACR in patients with lifestyle-related disease

	All patients			Non-diabetes mellitus			Diabetes mellitus		
uPCR (g/gCr)	*n*	median uACR (mg/gCr)	IQR	*n*	median uACR (mg/gCr)	IQR	*n*	median uACR(mg/gCr)	IQR
0.01	41	8.0	6.0–15.0	24	8.0	5.8–12.5	17	8.0	6.0–20.0
0.02	58	10.5	7.0–15.0	35	8.0	6.0–15.5	23	12.0	8.5–13.5
0.03	67	11.0	7.0–17.0	42	12.0	8.3–17.0	25	11.0	6.0–18.0
0.04	64	16.0	10.0–28.0	39	19.0	9.0–32.0	25	12.0	10.0–27.0
0.05	58	17.5	10 .3–29.0	35	18.0	10.5–27.0	23	17.0	11.0–27.5
0.06	65	21.0	12.0–37.0	43	19.0	11.5–36.5	22	25.0	14.0–36.8
0.07	56	29.5	13.0–41.0	37	28.0	12.0–35.0	19	41.0	25.0–47.0
0.08	36	26.0	18.0–53.3	25	25.0	14.0–53.0	11	32.0	22.0–45.5
0.09	40	39.0	24.8–57.3	27	43.0	25.0–57.5	13	32.0	23.0–43.0
0.10	28	51.0	35.8–63.0	22	48.0	36.5–58.8	6	63.0	42.0–66.8
0.11	26	60.5	44.3–73.5	16	68.0	58.3–77.3	10	46.0	42.3–54.8
0.12	29	57.0	34.0–78.0	18	59.0	46.3–79.3	11	52.0	32.5–71.5
0.13	23	71.0	55.0–92.0	13	79.0	55.0–101.0	10	64.0	55.8–77.8
0.14	27	67.0	40.0–88.0	18	78.5	39.0–104.3	9	51.0	48.0–74.0
0.15	25	71.0	48.0–104.0	18	72.5	50.3–104.8	7	69.0	52.5–88.0
0.16	25	95.0	53.0–120.0	13	116.0	79.0–133.0	12	80.0	51.8–98.5

*uACR* urinary albumin-to-creatinine ratio; *uPCR* urinary protein-to-creatinine ratio; *IQR* interquartile range

Regarding the number of knots in the linear spline of the uPCR and the median uACR, as well as the restricted cubic spline, we selected 4 knots, since the AIC values of the models using 4 knots were lower than those using 3 or 5 knots in the overall patients as well as in non-diabetic patients and were nearly equal in diabetic patients.

**Fig. 1:**
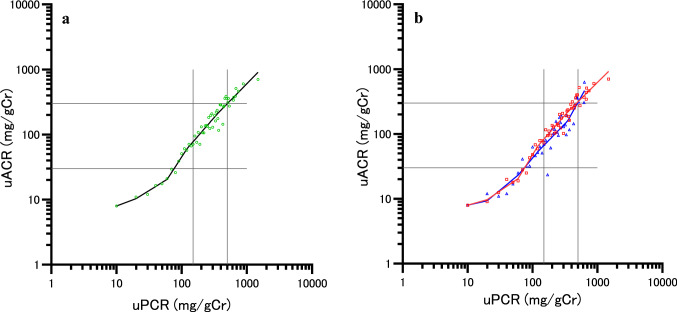
The associations between uPCR and median uACR by a linear spline using 4 knots. **a** non-diabetic and diabetic patients. Adjusted R-squared 0.965, AIC − 103.7. ○: Median uACR of non-diabetic and diabetic patients. **b** The red line, non-diabetics (Adjusted R-squared 0.967, AIC − 100.2); blue line, diabetics (Adjusted R-squared 0.899, AIC − 36.7). □: Median uACR of non-diabetic patients. △: Median uACR of diabetic patients. The 4 knots were at percentiles 5, 35, 65, 95 of 846 measurements, corresponding to uPCR of 20, 60, 120, and 350 mg/gCr. The two vertical lines indicate uPCR 150 and 500 mg/gCr, respectively, while the two horizontal lines indicate uACR 30 and 300 mg/gCr, respectively

Table 4 shows the equations to estimate the median uACR from the uPCR using 4-knots linear spline in the overall patients, in non-diabetic patients, and diabetic patients. Regarding the linear spline, the uPCR corresponding to the median uACR 30 mg/gCr was 0.077 g/gCr in the overall patients, while the median uACR corresponding to uPCR 0.15 g/gCr was 78 mg/gCr (Fig. 1a). In non-diabetic and diabetic patients, the uPCR corresponding to the median uACR30 mg/gCr was 0.075 and 0.075 g/gCr, respectively, while the median ACR corresponding to uPCR0.15 g/gCr was 83 and 67 mg/gCr (Fig. 1b), respectively. The median uACR corresponding to a uPCR of 0.50 g/gCr in the overall patients, non-diabetic patients, and diabetic patients was 297, 313, and 306 mg/gCr, respectively. When examining the shift of inclination in Δln (median uACR)/Δln (uPCR) before and after each knot, it increased significantly at PCR 0.060 g/gCr overall, with a significant increase in non-diabetics when looking at it by disease. Although it did increase in diabetics as well, it was not significant.

**Table 4:** Equations to estimate median uACR from uPCR using 4-knots linear spline and the change in slope before and after the range of the knots

uPCR (mg/gCr)	In patients with lifestyle-related disease	Non-diabetes and diabetes	Non-diabetes	Diabetes
ln(median uACR)	1R; < 20	0.3719 × ln(uPCR) + 1.2232	0.2801 × ln(uPCR) + 1.4345	0.2210 × ln(uPCR) + 1.5706
	2R; 20 to < 60	0.6224 × ln(uPCR) + 0.4727	0.6804 × ln(uPCR) + 0.2352	0.8277 × ln(uPCR) − 0.2471
	3R; 60 to < 120	1.5483 × ln(uPCR)-3.3183	1.6581 × ln(uPCR)-3.7678	1.1957 × ln(uPCR) − 1.7535
	4R; 120 to < 350	1.1524 × ln(uPCR)-1.4229	1.1401 × ln(uPCR)-1.2878	1.0335 × ln(uPCR) − 0.9770
	5R; 350 to < 500	1.0269 × ln(uPCR)-0.6880	0.9986 × ln(uPCR)-0.4592	1.8136 × ln(uPCR) − 5.5472

	CE	SE	*t*	*p* > *t*	95%CI	CE	SE	*t*	*p* > *t*	95%CI	CE	SE	*t*	*p* > *t*	95%CI
Change in slope															
1R vs 2R	0.25	0.52	0.48	0.64	− 0.80 to 1.30	0.40	0.52	0.77	0.45	− 0.65 to 1.45	0.61	0.86	0.70	0.49	− 1.14 to 2.36
2R vs 3R	0.93	0.38	2.43	0.02	0.16–1.69	0.98	0.38	2.58	0.01	0.21–1.74	0.37	0.63	0.58	0.56	− 0.91 to 1.65
3R vs 4R	− 0.40	0.29	− 1.38	0.17	− 0.97 to 0.18	− 0.52	0.29	− 1.81	0.08	− 1.09 to 0.06	-0.16	0.50	-0.33	0.75	− 1.17 to 0.85
4R vs 5R	− 0.13	0.18	-0.69	0.50	− 0.49 to 0.24	− 0.14	0.18	− 0.77	0.44	− 0.51 to 0.23	0.78	0.54	1.45	0.16	− 0.32 to 1.88

*uACR* urinary albumin-to-creatinine ratio; *uPCR* urinary protein-to-creatinine ratio, *ln* natural logarithm; *CE* coefficient; *SE* standard error; *CI* confidence interval, Unit of uPCR, mg/gCr; Unit of uACR, mg/gCr

**Fig. 2:**
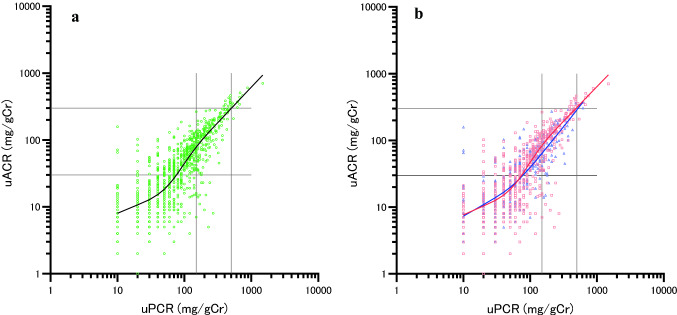
The associations between uPCR and median uACR by a restricted cubic spline using 4 knots. **a** non-diabetic and diabetic patients. Adjusted R-squared 0.966, AIC − 107.2. ○: uACR of non-diabetic and diabetic patients. **b** The red line, non-diabetics (Adjusted R-squared 0.967, AIC − 101.3); blue line, diabetics (Adjusted R-squared 0.899, AIC -38.4). □: uACR of non-diabetic patients. △: uACR of diabetic patients. The two vertical lines indicate uPCR 150 and 500 mg/gCr, respectively, while the two horizontal lines indicate uACR 30 and 300 mg/gCr, respectively

The relationship between the median uACR and uPCR was the same for the restricted cubic spline (Fig. 2 a,b).

The relationship between the uACR and the CO of the uPCR for determining whether it was greater or less than the uACR (Tables 5, 6).

**Table 5:** The analysis to discriminate uACR below and above each value in non-diabetic patients by the receiver operating characteristics curve (*n* = 591)

uACR (X mg/gCr)	*n*(≥ X mg/gCr)	AUC	AUC 95% CI	*p*	uPCR (g/Cr) CO(YI)	Sensitivity	specificity
10	493	0.827	0.789–0.865	< 0.0001	0.06	0.72	0.82
20	370	0.852	0.821–0.883	< 0.0001	0.09	0.67	0.92
30	314	0.878	0.850–0.906	< 0.0001	0.09	0.76	0.89
40	264	0.907	0.882–0.932	< 0.0001	0.09	0.85	0.87
50	233	0.922	0.898–0.945	< 0.0001	0.10	0.85	0.88
60	202	0.931	0.909–0.954	< 0.0001	0.11	0.87	0.89
70	176	0.948	0.929–0.967	< 0.0001	0.11	0.92	0.87
80	153	0.950	0.931–0.970	< 0.0001	0.12	0.92	0.86
90	142	0.956	0.938–0.974	< 0.0001	0.13	0.92	0.88
100	132	0.956	0.937–0.974	< 0.0001	0.13	0.92	0.87
110	113	0.959	0.944–0.975	< 0.0001	0.14	0.93	0.86
120	98	0.961	0.945–0.978	< 0.0001	0.15	0.92	0.87
130	87	0.962	0.944–0.980	< 0.0001	0.15	0.94	0.86
140	71	0.973	0.958–0.988	< 0.0001	0.16	0.96	0.87
150	65	0.975	0.960–0.991	< 0.0001	0.20	0.91	0.93
300	18	0.996	0.993–1.000	< 0.0001	0.39	1.00	0.98

*uACR* urinary albumin-to-creatinine ratio; *uPCR* urinary protein-to-creatinine ratio; *AUC* area under the curve CO(YI), cut-off value using Youden's index; *CI* confidence interval

**Table 6:** The analysis to discriminate uACR below and above each value in diabetic patients by the receiver operating characteristics curve (*n* = 318)

uACR (X mg/gCr)	n(≥ X mg/gCr)	AUC	AUC 95% CI	*p*	uPCR (g/Cr) CO(YI)	Sensitivity	specificity
10	276	0.820	0.768–0.872	*p* < 0.0001	0.06	0.66	0.88
20	208	0.862	0.821–0.903	*p* < 0.0001	0.06	0.81	0.84
30	169	0.889	0.851–0.926	*p* < 0.0001	0.07	0.83	0.83
40	137	0.887	0.848–0.927	*p* < 0.0001	0.10	0.76	0.90
50	110	0.892	0.851–0.934	*p* < 0.0001	0.10	0.84	0.86
60	93	0.888	0.843–0.933	*p* < 0.0001	0.10	0.85	0.81
70	80	0.889	0.842–0.937	*p* < 0.0001	0.12	0.83	0.83
80	65	0.909	0.861–0.957	*p* < 0.0001	0.13	0.86	0.85
90	56	0.905	0.851–0.960	*p* < 0.0001	0.15	0.84	0.89
100	50	0.895	0.834–0.955	*p* < 0.0001	0.16	0.80	0.89
110	40	0.912	0.845–0.979	*p* < 0.0001	0.16	0.90	0.88
120	36	0.914	0.842–0.986	*p* < 0.0001	0.16	0.92	0.87
130	32	0.914	0.833–0.994	*p* < 0.0001	0.20	0.91	0.94
140	28	0.901	0.810–0.993	*p* < 0.0001	0.20	0.89	0.93
150	23	0.942	0.867–1.018	*p* < 0.0001	0.21	0.96	0.93
300	5	1.000	1.000–1.000	0.00012	0.48	1.00	1.00

*uACR* urinary albumin-to-creatinine ratio; *uPCR* urinary protein-to-creatinine ratio; *AUC* area under the curve CO(YI), cut-off value using Youden's index; *CI* confidence interval

The uPCR that differentiated the uACR (for uACR 10–300 mg/gCr) was examined using an ROC curve for a total of 591 measurements in patients with non-diabetic lifestyle-related diseases and a total of 318 measurements in patients with diabetes. For uACR 10–300 mg/gCr, the higher the uACR value, the higher the AUC of the ROC curve, the CO of uPCR, the sensitivity, and the specificity. A uACR of greater or less than 30 mg/gCr was differentiated with a uPCR of 0.09 g/gCr in patients with non-diabetic lifestyle diseases (AUC 0.88, Sn 0.76, Sp 0.89), and a uPCR of 0.07 g/gCr in patients with diabetes (AUC 0.89, Sn 0.83, Sp 0.83). The CO of uPCR 0.15 g/gCr, according to the Youden’s index, differentiated a uACR of greater or less than 120 mg/gCr in patients with non-diabetic lifestyle diseases (AUC 0.96, Sn 0.92, Sp 0.87), and uACR greater or less than 90 mg/gCr for diabetes (AUC 0.91, Sn 0.84, Sp 0.89).

**Fig. 3:**
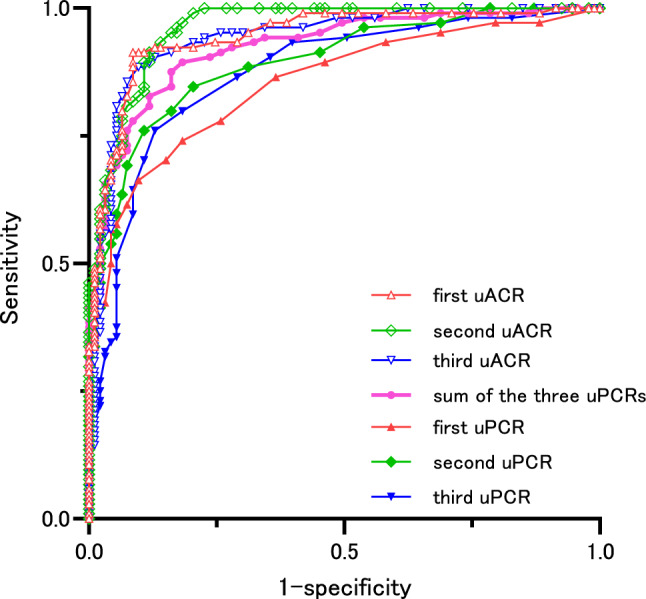
Differentiation of patients with normoalbuminuria and micro- and macroalbuminuria by the the first uACR(△), second uACR (◇), third uACR (▽), first uPCR (▲), second uPCR (◆), third uPCR (▼), and sum of the three uPCR values (●), according to an ROC analysis

Differentiation of microalbuminuria by the uPCR and uACR in non-diabetic patients (Fig. 3, Table 7).

**Table 7:** Cut-off value (YI) of the uPCR and uACR distinguishing patients with normoalbuminuria (*n* = 93) and micro- and macroalbuminuria (*n* = 104) in non-diabetic patients

Test variables	1uPCR	2uPCR	3uPCR	1.2uPCR	2.3uPCR	1.3uPCR	1.2.3uPCR	1uACR	2uACR	3uACR
ROC curve area	0.854	0.894	0.873	0.912^a^	0.911^b^	0.904^c^	0.924^d^	0.945^e^	0.961^f^	0.941^ g^
Standard error	0.027	0.022	0.026	0.020	0.021	0.021	0.018	0.01598	0.012	0.017
*p* value	< 0.0001	< 0.0001	< 0.0001	< 0.0001	< 0.0001	< 0.0001	< 0.0001	< 0.0001	< 0.0001	< 0.0001
CO(YI)^h^	0.10	0.09	0.09	0.16	0.16	0.17	0.23	31	31	30
Sensitivity	0.66	0.76	0.76	0.85	0.85	0.84	0.88	0.91	0.91	0.89
Specificity	0.82	0.89	0.87	0.87	0.86	0.85	0.84	0.91	0.88	0.90

1, 2, 3 uPCR denote first, second, and third uPCR, respectively. 1.2uPCR, 2.3uPCR, 1,3uPCR each denote the sum of the two indicated uPCRs. 1.2.3uPCR denotes the sum of the three uPCRs. 1, 2, 3uACR denotes first, second, and third uACR, respectively

uACR, urinary albumin-to-creatinine ratio; uPCR, urinary protein-to-creatinine ratio, CO(YI), cut-off value using Youden's index by the receiver operating characteristics curve

^a^vs. 1uPCR, *p* = 0.0005

^b^vs. 3uPCR, *p* = 0.005

^c^vs 3uPCR, *p* = 0.047

^d^vs. 1uPCR, *p* = 0.0005; vs. 2uPCR, *p* = 0.047; vs. 3uPCR, *p* = 0.003

^e^vs. 1uPCR, *p* = 0.0002; vs. 2uPCR, *p* = 0.045; vs. 3uPCR, *p* = 0.020

^f^vs. 1uPCR, *p* = 0.0002; vs. 2uPCR, *p* = 0.001; vs. 3uPCR, *p* = 0.001; vs. 1.2UP, *p* = 0.018, vs. 2.3UP, *p* = 0.015; vs. 1.3UP, *p* = 0.013

^g^vs. 1uPCR, *p* = 0.004; vs. 3uPCR, *p* = 0.002

^h^Unit of uPCR, g/gCr; Unit of uACR, mg/gCr

A single uPCR was useful for differentiating normoalbuminuria and micro- and macroalbuminuria in both non-diabetics and diabetics. The CO of the second uPCR measurement was 0.09 g/gCr in non-diabetics (AUC 0.89, Sn 0.76, Sp 0.89) and 0.06 g/gCr in diabetics (AUC 0.83, Sn 0.86, Sp 0.69). The sum of the uPCRs measured twice, at the first and second, as well as the second and third measurements, was useful for the differentiation of micro- and macroalbuminuria in non-diabetics (AUC 0.91, CO 0.16 g/gCr). The sum of the three uPCRs was useful for differentiating microalbuminuria (AUC 0.92, CO uPCR 0.23 g/gCr, Sn 0.88, Sp 0.84) and was significantly more useful than the single uPCR. The second uACR was useful for differentiating microalbuminuria (AUC 0.96, CO uACR 31 mg/gCr, Sn 0.91, Sp 0.88) and significantly more useful than a single uPCR or the sum of two uPCRs; it also tended to be useful for comparing the sum of three uPCRs; however, no significant difference was found (*p* = 0.059). There was no significant difference when the first, second, and third uACR were directly compared in the differentiation of microalbuminuria. However, while the second uACR was more useful for differentiating microalbuminuria than the sum of proteinuria measured twice, the first and third uACR measurements did not differ to a statistically significant extent, with the sum of proteinuria measured twice.

Factors affecting CO of the uPCR that differentiates a uACR of greater or less than 30 mg/gCr, or microalbuminuria (Tables 8, 9).

**Table 8:** Cut-off value (YI) of uPCR discriminating uACR ≥ 30 mg/gCr in patients with or without diabetes mellitus according to the age, sex, GFR category (*n* = 591)

Cause	Non-diabetes mellitus						
Test variables		Age (years)		Sex		GFR category	
	< 65	65 to < 80	≥ 80	Male	Female	G1-3a	G3b-4
*n*	201	228	162	372	219	375	216
*n* (uACR ≥ 30 mg/gCr)	108	108	98	187	127	185	129
ROC curve area	0.896	0.862	0.863	0.861	0.908	0.865	0.892
Standard error	0.022	0.026	0.030	0.020	0.021	0.020	0.022
*p* value	< 0.0001	< 0.0001	< 0.0001	< 0.0001	< 0.0001	< 0.0001	< 0.0001
CO(YI) (g/gCr)	0.09	0.09	0.08	0.09	0.09	0.09	0.10
Sensitivity	0.74	0.72	0.84	0.74	0.78	0.72	0.79
Specificity	0.91	0.92	0.80	0.87	0.94	0.91	0.89

Cause	Diabetes mellitus						
Test variables		Age (years)		Sex		GFR category	
	< 65	65 to < 80	≥ 80	Male	Female	G1-3a	G3b-4
*n*	123	153	42	210	108	249	69
*n* (uACR ≥ 30 mg/gCr)	56	81	32	105	64	117	52
ROC curve area	0.879	0.889	0.878	0.909	0.842	0.872	0.912
Standard error	0.033	0.029	0.069	0.022	0.037	0.025	0.036
*p* value	< 0.0001	< 0.0001	0.0004	< 0.0001	< 0.0001	< 0.0001	< 0.0001
CO(YI) (g/gCr)	0.06	0.07	0.08	0.07	0.11	0.07	0.07
Sensitivity	0.89	0.83	0.84	0.88	0.63	0.80	0.89
Specificity	0.76	0.85	0.90	0.87	1.00	0.84	0.77

*uACR* urinary albumin-to-creatinine ratio; *uPCR* urinary protein-to-creatinine ratio, *CO(YI)* cut-off value using Youden's index; *GFR* glomerular filtration rate

**Table 9:** Cut-off value (YI) of 2nd uPCR discriminating normoalbuminuria and micro- and macroalbuminuria in patients with or without diabetes mellitus according to the age, sex, GFR category

	Non-diabetes mellitus							
Test variables			Age (years)		Sex		GFR category	
	All	< 65	65 to < 80	≥ 80	Male	Female	G1-3a	G3b-4
*n*	197	67	76	54	124	73	125	72
*n* (≥ microalbuminuria)	104	35	36	33	61	43	61	43
ROC curve area	0.894	0.949	0.876	0.829	0.893	0.902	0.917	0.840
Standard error	0.022	0.025	0.039	0.058	0.028	0.035	0.023	0.047
*P* value	< 0.0001	< 0.0001	< 0.0001	< 0.0001	< 0.0001	< 0.0001	< 0.0001	< 0.0001
CO(YI) (g/gCr)	0.09	0.09	0.09	0.07	0.09	0.09	0.07	0.10
Sensitivity	0.76	0.83	0.69	0.85	0.77	0.74	0.85	0.74
Specificity	0.89	0.97	0.90	0.71	0.87	0.93	0.88	0.83

	Diabetes mellitus							
Test variables			Age (years)		Sex		GFR category	
	All	< 65	65 to < 80	≥ 80	Male	Female	G1-3a	G3b-4
*n*	106	41	51	14	70	36	83	23
*n* (≥ microalbuminuria)	55	19	25	11	33	22	39	16
ROC curve area	0.831	0.835	0.812	0.879	0.845	0.807	0.786	0.951
Standard error	0.040	0.065	0.062	0.100	0.049	0.072	0.051	0.043
*p* value	< 0.0001	0.0003	0.0001	0.0516	< 0.0001	0.0022	< 0.0001	0.0007
CO(YI) (g/gCr)	0.06	0.06	0.10	0.15	0.06	0.12	0.06	0.07
Sensitivity	0.86	0.90	0.68	0.73	0.91	0.59	0.80	1.00
Specificity	0.69	0.73	0.88	1.00	0.68	0.93	0.68	0.71

Microalbuminuria: urinary albumin-to-creatinine ratio 30–299 mg/gCr at least twice for 3 measurements

*uPCR* urinary protein-to-creatinine ratio, *CO(YI)* cut-off value using Youden's index; *GFR* glomerular filtration rate

When non-diabetics and diabetics were divided according to age, sex, or GFR category, the uPCR was useful for distinguishing between a uACR of greater or less than 30 mg/gCr or microalbuminuria in each respective division. While no effects of age or sex on the CO of uPCR were found in non-diabetics, the CO tended to be low in non-elderly people and in male diabetes patients. The effects of the GFR category were seen in non-diabetics and the CO in G1-3a tended to be lower than that in G3b-4.

The sensitivity, specificity and Youden’s index for discriminating non-diabetic patients with a uACR of ≥ 30 mg/gCr or micro- and macroalbuminuria by the uPCR according to GFR category are shown in Table 10. For uPCR 0.07, 0.09 and 0.15 g/gCr, the Youden’s index of the CO for differentiating microalbuminuria was the largest at uPCR 0.09 g/gCr in G1-4 and G3b-4, and uPCR 0.07 g/gCr in G1-3a.

**Table 10:** Sensitivity, specificity and Youden,s index for discriminating non-diabetic patients with uACR ≥ 30 mg/gCr or micro- and macroalbuminuria by uPCR according to GFR category

GFR category	Albuminuria category			uPCR(g/gCr)	
			≥ 0.07	≥ 0.09	≥ 0.15
G1-4	uACR ≥ 30 mg/gCr	Sensitivity	0.84	0.76	0.47
		Specificity	0.77	0.89	0.98
		Youden's Index	0.61	0.65	0.44
G1-4	≥ Microalbuminuria	Sensitivity	0.85	0.76	0.50
		Specificity	0.80	0.89	0.98
		Youden's Index	0.64	0.65	0.48
G1-3a	uACR ≥ 30 mg/gCr	Sensitivity	0.82	0.72	0.46
		Specificity	0.81	0.91	0.97
		Youden's Index	0.62	0.64	0.43
G1-3a	≥ Microalbuminuria	Sensitivity	0.85	0.75	0.54
		Specificity	0.88	0.95	1.00
		Youden's Index	0.73	0.71	0.54
G3b-4	uACR ≥ 30 mg/gCr	Sensitivity	0.88	0.80	0.47
		Specificity	0.68	0.85	0.98
		Youden's Index	0.55	0.65	0.45
G3b-4	≥ Microalbuminuria	Sensitivity	0.84	0.77	0.44
		Specificity	0.62	0.76	0.93
		Youden's Index	0.46	0.53	0.37

Microalbuminuria: urinary albumin-to-creatinine ratio 30–299 mg/gCr at least twice for 3 measurements

*uACR* urinary albumin-to-creatinine ratio; *uPCR* urinary protein-to-creatinine ratio; *GFR* glomerular filtration rate

When the CO that differentiated uACR ≥ 30 mg/gCr was reduced from uPCR 0.15 g/gCr to uPCR 0.09 g/gCr, all 9 cases with a uACR of 100–299 mg/gCr in G1-3a and 8 of the 10 cases with a uACR of 100–299 mg/gCr in G3b-4 were newly determined as uACR ≥ 30 mg/gCr (Table 10). Out of 32 false positives associated with a CO of uPCR 0.07 g/gCr in G1-3a, the uACR was ≥ 10 mg/gCr in 23 cases (71.8%), while all 11 false positives associated with a uPCR of 0.09 g/gCr in G3b-4 had a uACR of ≥ 10 mg/gCr (Table 11).

**Table 11:** Distribution of uACR according to GFR category and uPCR in non-diabetic patients

		uPCR (g/gCr)					
GFR category	uACR(mg/gCr)	< 0.07	0.07 to < 0.15	< 0.09	0.09 to < 0.15	0.15 to < 0.50	≥ 0.50
G1-3a	0–29	153	32	173	12	5	0
	0–9	59	9	64	4	0	0
	10–19	70	11	78	3	4	0
	20–29	24	12	31	5	1	0
G1-3a	30–299	34	66	51	49	75	1
	30–59	28	32	40	20	7	0
	60–99	6	25	11	20	12	0
	100–149	0	8	0	8	28	0
	150–199	0	1	0	1	12	0
	200–299	0	0	0	0	16	1
G1-3a	≥ 300	0	0	0	0	6	3
G3b-4	0–29	59	26	74	11	2	0
	0–9	26	3	29	0	1	0
	10–19	23	15	32	6	0	0
	20–29	10	8	13	5	1	0
G3b-4	30–299	16	52	26	42	52	0
	30–59	12	28	21	19	5	0
	60–99	2	16	3	15	9	0
	100–149	2	7	2	7	22	0
	150–199	0	1	0	1	7	0
	200–299	0	0	0	0	9	0
G3b-4	≥ 300	0	0	0	0	3	6

*GFR* glomerular filtration rate; *uACR* urinary albumin-to-creatinine ratio; *uPCR* urinary protein-to-creatinine ratio

**Fig. 4:**
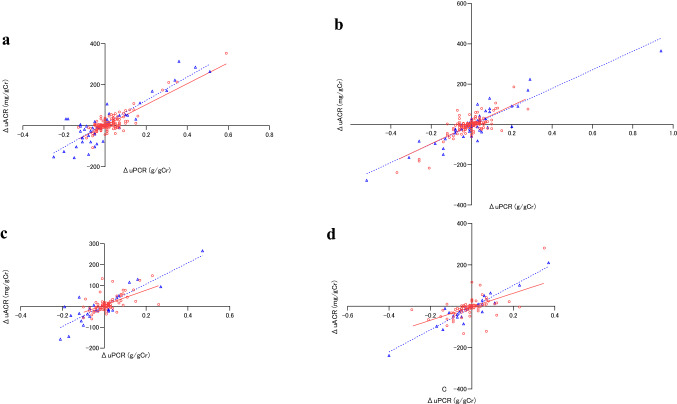
(a) Association of differences between the second and first uPCR value and the uACR in non-diabetic patients. ○: first uPCR < 0.15 g/gCr, —: ΔuACR = 510.4 × ΔuPCR + 0.4, *R* = 0.85, *p* = 6.5 × 10^–44^. △: first uPCR ≥ 0.15 g/gCr, ---: ΔuACR = 569.9 × ΔuPCR + 9.2, *R* = 0.88, *p* = 3.1 × 10^–16^. **b** Association of differences between the third and second uPCR value and the uACR in non-diabetic patients. ○: first uPCR < 0.15 g/gCr, —: ΔuACR = 473.1 × ΔuPCR-1.5, *R* = 0.76, *p* = 3.8 × 10^–29^. △: first uPCR ≥ 0.15 g/gCr, ---: ΔuACR = 460.6 × ΔuPCR-5.4, R = 0.90, p = 4.1 × 10^–18^. **c** Association of differences between the second and first uPCR value and the uACR in diabetic patients. ○: first uPCR < 0.15 g/gCr, —: ΔuACR = 353.9 × ΔuPCR + 6.8, *R* = 0.59, *p* = 8.1 × 10^–9^. △: first uPCR ≥ 0.15 g/gCr, ---: ΔuACR = 507.1 × ΔuPCR + 5.3, R = 0.88, p8.7 =  × 10^–9^. **d** Association of differences between the third and second uPCR value and the uACR in diabetic patients. ○: first uPCR < 0.15 g/gCr, —: ΔuACR = 326.1 × ΔuPCR-2.6, *R* = 0.57, *p* = 2.7 × 10^–8^. △: first uPCR ≥ 0.15 g/gCr, ---: ΔuACR = 536.4 × ΔuPCR-6.5, *R* = 0.94, *p* = 1.1 × 10^–12^

When examining the correlation of changes in the uACR and uPCR, after dividing the first uPCR in non-diabetics and diabetics into greater than and less than 0.15 g/gCr, a positive correlation was confirmed with respect to the change of 2–1 times and 3–2 times when the first uPCR was less than and greater than 0.15 g/gCr (Fig. 4).

**Fig. 5:**
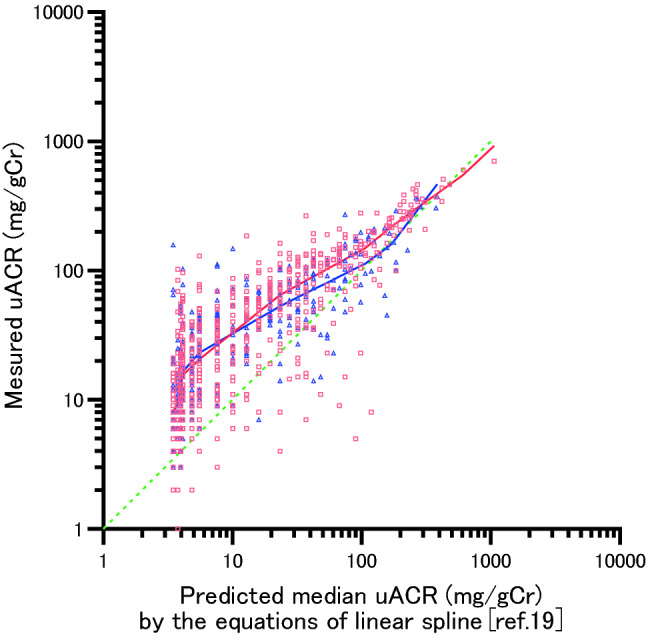
The scatterplot of measured uACR in this study and predicted median uACR from the equations of linear spline in reference 19. The dotted line indicate the line of identity. Red curve indicates the predicted median uACR in non-diabetic patients and blue curve indicates the predicted median uACR in diabetic patients from linear spline model of this study. The measured uACRs are above the line of identity in the low predicted uACR in reference 19. The predicted median uACRs in this study became approximately equal to the predicted value in reference 19 at higher levels of uACR. □: uACR of non-diabetic patients. △: uACR of diabetic patients

Figure 5 indicates the predicted median ACR (mg/gCr) according to the equation from the linear spline from the uPCR by Weaver et al. [19] and the scatterplot of the measured ACR (mg/gCr) based on this study. The measured uACR did not distribute symmetrically along the dotted line of identity, with the low predicted median ACR distributed on the upper part of the dotted line. The red and blue lines indicate the predicted median ACR from the uPCR according to the linear spline in the non-diabetics and diabetics in this study, respectively. The predicted median uACR of 30 mg/gCr, determined by the equation of Weaver et al. [19] corresponded to 74 mg/gCr for non-diabetics and 60 mg/gCr for diabetics, as determined by the equation of this study, while the predicted mean uACR of 100 mg/gCr corresponded to 144 mg/gCr for non-diabetics and 109 mg/gCr for diabetics. Regarding the low predicted median uACR value, while the predicted median uACR value of this study was higher than that determined by the equation of Weaver et al., there was a tendency for the gap to become smaller as the uACR increased, with the values ultimately matching.

### The intraday and interday variance of uPCR and uACR of the same sample

The study on the intra- and interday variance of uPCR and uACR included 88 subjects: age 73.1 ± 12.7 years (mean ± SD); 48 males; and 40 females. Their underlying lifestyle-related disease was 65 cases of non-diabetic disease and 23 cases of diabetes. Regarding the G stage, there were 2 cases of G1, 18 cases of G2, 29 cases of G3a, 29 cases of G3b, and 10 cases of G4.

**Fig. 6:**
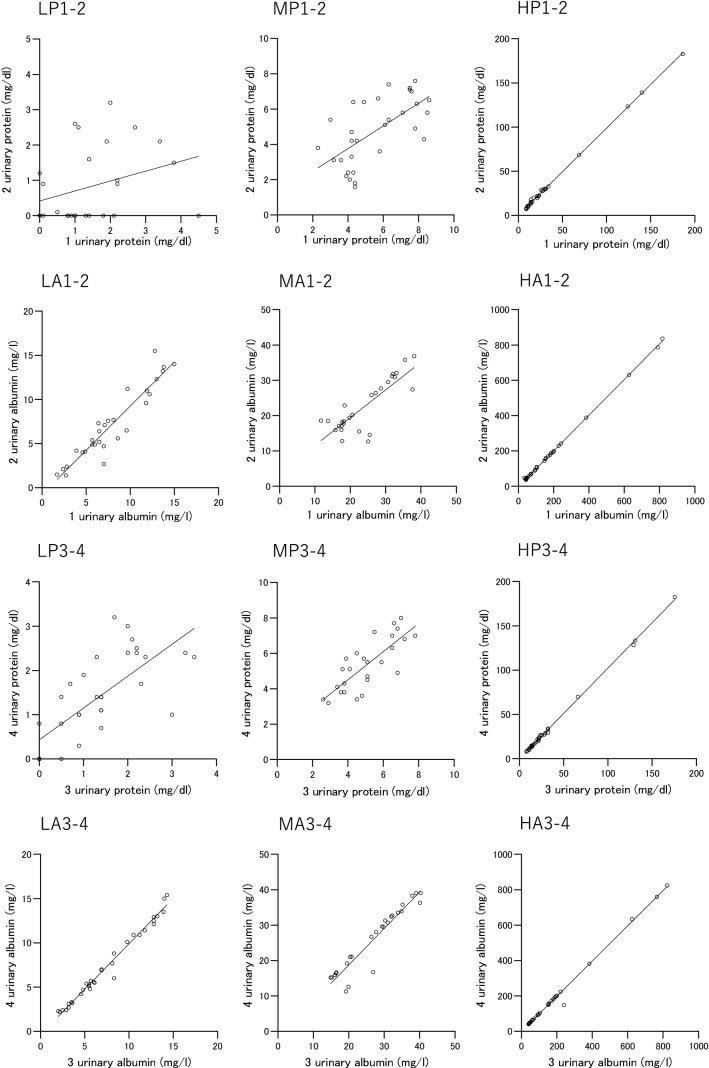

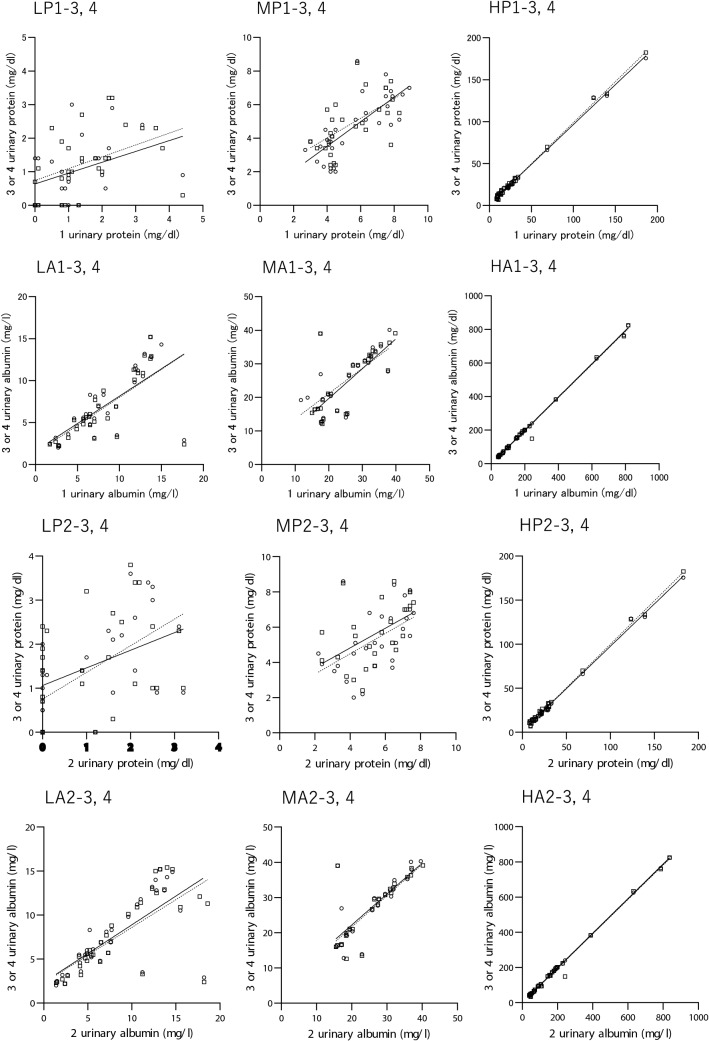
Intraday and interday correlation of urinary protein and albumin of the same sample according to the mean of each 2 levels. The symbols in the upper left of the figure correspond to the symbols in Tables 12, 13, 14. ○, correlations between 1st and 2nd, 3rd and 4th, 1st and 3rd, and 2nd and 3rd measurements; □, correlations between 1st and 4th, and 2nd and 4th measurements

The mean values of two urinary protein measurements were categorized into three groups: less than 3 mg/dl (hereinafter, the “low urinary protein concentration group”); 3 mg/dl or more but less than 8 mg/dl (hereinafter, the “medium urinary protein concentration group”); and 8 mg/dl or more (hereinafter, the "high urinary protein concentration group”) (Table 12, Fig. 6).

**Table 12:** Intraday and interday correlation and variance of urinary protein of the same sample according to the mean urinary protein level

	Mean of 2 urinary protein			Mean of 2uCr/sCr	Mean of 2uPCR	Mean of 2uACR	Regression line	*r*	*p*	Bias of uP
	Range (mg/dl)	n	(mg/dl)		(g/gCr)	(g/gCr)				(mg/dl)
Intraday										
3-4 h										
LP1-2	< 3.0	27	0.7 (0.4–1.8)	45.1 (36.8–79.1)	0.02 (0.01–0.03)	12.5 (8.3–28.0)	2uP = 0.2810 × 1uP + 0.4184	0.31	0.1139	− 0.8 (− 1.3 to 0.1)
MP1-2	3.0 to < 8.0	31	5.4 (3.4–6.7)^a2^	52.7 (35.7–88.8)	0.10 (0.05–0.12)^a2^	41.5 (18.0–64.5)^a2^	2uP = 0.6424 × 1uP + 1.1910	0.64	0.0001	− 0.9 (− 2.0 to − 0.1)
HP1-2	≥ 8.0	30	20.9 (11.9–29.2)^a2b2^	71.0 (50.2–119.5)^a1^	0.23 (0.12–0.40)^a2b2^	148.5 (38.9–265.9)^a2b2^	2uP = 0.9875 × 1uP + 0.1011	0.99	< 0.0001	− 0.8 (− 1.1 to 0.1)
3 min										
LP3-4	< 3.0	32	1.3 (0.6–2.2)	44.4 (37.2–69.5)	0.03 (0.01–0.05)	12.5 (8.5–24.3)	4uP = 0.7195 × 3uP + 0.4324	0.73	< 0.0001	0.0 (− 0.3 to 0.5)
MP3-4	3.0 to < 8.0	26	5.1 (4.1–6.4)^a2^	56.5 (32.7–99.9)	0.10(0.06–0.13)^a2^	46.3 (28.6–94.8)^a2^	4uP = 0.7962 × 3uP + 1.318	0.79	< 0.0001	0.5 (− 0.4 to 1.0)
HP3-4	≥ 8.0	30	21.3 (13.8–28.8)^a2b2^	76.2 (49.1–128.9)^a2^	0.21 (0.11–0.39)^a2b2^	150.8 (34.8–270.0)^a2b1^	4uP = 1.025 × 3uP − 0.2500	0.99	< 0.0001	0.3 (− 0.5 to 1.4)
Interday										
3–4 days										
LP1-3	< 3.0	26	0.9(0.7–1.7)	45.9 (36.5–67.4)	0.03 (0.02–0.04)	14.8 (9.1–28.5)	3uP = 0.3230 × 1uP + 0.6355	0.39	0.0504	− 0.3 (− 0.8 to 0.2)
MP1-3	3.0 to < 8.0	32	4.4 (3.5–6.7)^a2^	53.8 (35.9–97.8)	0.08(0.06–0.13)^a2^	42.3 (15.5–72.9)^a2^	3uP = 0.7320 × 1uP + 0.5866	0.75	< 0.0001	− 1.0 (− 1.9 to −0.1)
HP1-3	≥ 8.0	30	21.4 (13.1–29.0)^a2b2^	73.3 (49.5–127.8)^a2^	0.22 (0.12–0.39)^a2b2^	150.3 (35.9–267.3)^a2b2^	3uP = 0.9502 × 1uP + 1.505	0.99	< 0.0001	0 (− 1.0 to 1.6)
LP1-4	< 3.0	29	1.4 (0.7–2.1)	47.1 (37.3–71.1)	0.03 (0.02–0.04)	13.0(8.5–24.0)	4uP = 0.3528 × 1uP + 0.7391	0.43	0.0198	− 0.1 (− 0.8 to 0.7)
MP1-4	3.0 to < 8.0	27	5.2(3.8–6.7)^a2^	42.1 (33.8–98.8)	0.09 (0.06–0.13)^a2^	46.5 (21.8–81.5)^a2^	4uP = 0.5880 × 1uP + 1.660	0.59	0.0013	− 0.5 (− 1.7 to 0.1)
HP1-4	≥ 8.0	32	19.8 (12.0–27.7)^a2b2^	73.3(50.8–128.1)^a1^	0.20 (0.11–0.36)^a2b2^	128.0 (34.9–238.3)^a2b1^	4uP = 0.9775 × 1uP + 1.063	0.99	< 0.0001	0.2 (− 0.8 to2.5)
3–4 days										
LP2-3	< 3.0	32	1.1 (0.3–1.9)	44.4 (36.4–71.3)	0.02 (0.01–0.04)	14.3 (8.9–27.1)	3uP = 0.6062 × 2uP + 0.7511	0.63	0.0001	0.5 (0 to 0.9)
MP2-3	3.0 to < 8.0	28	5.3 (4.2–6.5)^a2^	56.6(35.8–97.6)	0.09 (0.06–0.11)^a2^	43.8 (20.3–66.8)^a2^	3uP = 0.5769 × 2uP + 2.179	0.52	0.0049	− 0.2 (− 1.2 to 0.7)
HP2-3	≥ 8.0	28	21.4 (14.1–30.1)^a2b2^	73.6 (51.0–129.0)^a2^	0.25 (0.12–0.42)^a2b2^	166.8 (39.9–286.1)^a2b2^	3uP = 0.9616 × 2uP + 1.437	0.99	< 0.0001	0.3 (− 1.4 to 2.3)
LP2-4	< 3.0	33	1.2 (0.6–2.1)	46.9 (37.3–79.5)	0.02 (0.01–0.04)	12.5 (7.5–23.0)	4uP = 0.4003 × 2uP + 1.060	0.41	0.0169	0.5 (− 0.1 to 1.4)
MP2-4	3.0 to < 8.0	27	5.8(4.4–6.9)^a2^	52.9(33.4–88.9)	0.10 (0.07–0.13)^a2^	46.5 (24.3–87.0)^a2^	4uP = 0.5583 × 2uP + 2.587	0.50	0.0082	− 0.2 (− 1.3 to 1.1)
HP2-4	≥ 8.0	28	21.8 (14.5–29.6)^a2b2^	73.6 (50.9–129.1)^a1^	0.24 (0.12–0.43)^a2b2^	166.3 (39.8–288.1)^a2b1^	4uP = 0.9875 × 2uP + 1.157	0.99	< 0.0001	0.1 (− 0.4 to 2.7)

The numbers of 1, 2, 3, and 4 represent the first, second, third, and fourth measurements, respectively, of urinary protein, albumin, and creatinine. Data are presented as the median (IQR)

*uCr/sCr* urine-to-serum creatinine ratio; *uP* urinary protein; *uACR* urinary albumin-to-creatinine ratio; *uPCR* urinary protein-to-creatinine ratio; *LP* low level of urinary protein; *MP* medium level of urinary protein; *HP* high level of urinary protein

^a1^*p* < 0.05 vs. LP

^a2^*p* < 0.01 vs. LP

^b1^*p* < 0.05 vs. MP

^b2^*p* < 0.01 vs. MP by the Steel–Dwass test; bias, difference of the later measurement against the previous measurement

The uCr/sCr in the low urinary protein concentration group was lower than the high urinary protein concentration group (*p* < 0.05). Among the three groups, uPCR and uACR were significantly different. The median of uPCR and uACR was, respectively, 0.02 to 0.03 g/gCr, 13–15 mg/gCr in the low urinary protein concentration group, 0.08–0.10 g/gCr, 42–47 mg/gCr in the medium urinary protein concentration group, and 0.21–0.25 g/gCr, 128–166 mg/gCr in the high urinary protein concentration group (Table 12).

Regarding the intraday variance of urinary protein in the low urinary protein concentration group, no correlation was found between the first and second measurements with an interval of 3–4 h of refrigeration, while a significant positive correlation was noted between the third and fourth consecutive measurements 3–4 days later. Positive interday correlations were found between the first and fourth, second and third, and second and fourth measurements, but not between the first and third measurements. Both the intraday and interday correlations were positive and significant in the medium urinary protein concentration group. In the high urinary protein concentration group, the intraday and interday correlations were positive, significant, and even stronger.

The mean values of two measurements of albuminuria were categorized into three groups: less than 15 mg/dl (hereinafter, the “low urinary albumin concentration group”); 15 mg/dl or more but less than 40 mg/dl (hereinafter, the “medium urinary albumin concentration group”); and 40 mg/dl or more (hereinafter, the “high urinary albumin concentration group”) (Table 13, Fig. 6). The uACR was also different among the three groups categorized according to the urinary albumin concentration. The median of uACR was 10–12 mg/gCr and 37–41 mg/gCr in the low and medium urinary albumin concentration groups, respectively (Table 13). The two intraday comparisons and four interday comparisons all indicated significant positive correlations in all groups categorized by albuminuria.

**Table 13:** Intraday and interday correlation and variance of the urinary albumin of the same sample according to the maen urinary albumin level

	Mean of 2 urinary albumin			Mean of 2uACR	Regression line	*r*	*p*	Bias of uA
	Range (mg/l)	*n*	(mg/l)	(mg/gCr)				(mg/l)
Intraday								
3-4 h								
LA1-2	< 15.0	29	6.9 (4.9–10.7)	12.0 (7.5–19.5)	2uA = 1.002 × 1uA − 0.7668	0.94	< 0.0001	− 0.6 (− 1.3 to − 0.1)
MA1-2	15.0 to < 40.0	27	20.1 (17.6–30.8)^a2^	36.9 (21.8–54.0)^a2^	2uA = 0.7850 × 1uA + 3.729	0.83	< 0.0001	− 0.5 (− 1.4 to 0.1)
HA1-2	≥ 40.0	32	99.9 (46.0–188.7)^a2b2^	153.0 (59.9–234.6)^a2b2^	2uA = 1.010 × 1uA-1.479	0.99	< 0.0001	− 0.7 (− 2.4 to 0.7)
3 min								
LA3-4	< 15.0	33	5.8 (4.4–11.1)	9.5 (8.0–16.5)	4uA = 1.028 × 3uA − 0.3809	0.99	< 0.0001	− 0.2 (− 0.4 to 0.1)
MA3-4	15.0 to < 40.0	26	28.7 (17.3–33.4)^a2^	41.3 (27.9–59.0)^a2^	4uA = 1.029 × 3uA − 1.851	0.95	< 0.0001	0 (− 0.4 to 0.3)
HA3-4	≥ 40.0	29	104.6 (53.9–194.2)^a2b2^	168.0 (76.0–285.0)^a2b2^	4uA = 0.995 × 3uA − 1.396	0.99	< 0.0001	0.7 (− 0.4 to 1.9)^a1^
Interday								
3–4 days								
LA1-3	< 15.0	30	6.9 (5.2–10.7)	12.0 (8.3–21.7)	3uA = 0.6524 × 1uA + 1.624	0.71	< 0.0001	− 0.6 (− 1.7 to 0.1)
MA1-3	15.0 to < 40.0	27	22.3 (17.8–31.6)^a2^	39.5 (20.8–54.8)^a2^	3uA = 0.7534 × 1uA + 6.078	0.65	< 0.0001	0.2 (− 3.0 to 1.6)
HA1-3	≥ 40.0	31	98.9 (47.8–191.6)^a2b2^	159.0 (70.5–252.5)^a2b2^	3uA = 0.9927 × 1uA − 0.8079	0.99	< 0.0001	− 0.7 (− 2.7 to 0.3)
LA1-4	< 15.0	31	6.5(5.2–11.2)	11.5(8.3–20.9)	4uA = 0.6660 × 1uA + 1.343	0.71	< 0.0001	− 0.7(− 1.8 to − 0.3)
MA1-4	15.0 to < 40.0	27	26.4(17.9–32.5)^a2^	39.5 (21.3–55.3)^a2^	4uA = 0.8901 × 1uA + 1.851	0.77	< 0.0001	− 0.2 (− 3.4 to 0.7)
HA1-4	≥ 40.0	30	101.7 (50.9–194.0)^a2b2^	162.8 (79.0–268.8)^a2b2^	4uA = 0.9980 × 1uA − 2.156	0.99	< 0.0001	− 0.5(− 2.4 to 1.3)
3–4 days								
LA2-3	< 15.0	33	6.8(5.1–11.4)	11.5 (8.0–22.7)	3uA = 0.6530 × 2uA + 2.363	0.72	< 0.0001	0.5 (− 0.2 to 0.9)
MA2-3	15.0 to < 40.0	25	27.5 (19.0–31.5)^a2^	38.0 (21.5–53.5)^a2^	3uA = 0.8774 × 2uA + 4.663	0.78	< 0.0001	0.7 (0.3 to 1.50)
HA2-3	≥ 40.0	30	103.1 (50.1–192.7)^a2b2^	162.0 (68.1–267.1)^a2b2^	3uA = 0.9830 × 2uA + 0.6598	0.99	< 0.0001	− 0.1 (− 4.9 to 1.3)
LA2-4	< 15.0	35	7.3 (4.9–12.7)	11.5 (7.5–22.8)	4uA = 0.6301 × 2uA + 2.294	0.73	< 0.0001	0.4 (− 0.7 to 0.7)
MA2-4	15.0 to < 40.0	23	27.7 (18.5–32.0)^a2^	40.5(24.3–58.3)^a2^	4uA = 0.8886 × 2uA + 3.865	0.79	< 0.0001	0.8 (− 0.2 to 1.4)
HA2-4	≥ 40.0	30	102.8 (50.6–194.1)^a2b2^	161.3 (54.3–268.6)^a2b2^	4uA = 0.9783 × 2uA − 0.6403	0.99	< 0.0001	1.3 (− 4.8 to 2.8)

The numbers of 1, 2, 3, and 4 represent the first, second, third, and fourth measurements, respectively, of urinary albumin, and creatinine. Data are presented as the median (IQR)

*uA* urinary albumin; *uACR* urinary albumin-to-creatinine ratio; *uPCR* urinary protein-to-creatinine ratio; *LA* low level of urinary albumin; *MA* medium level of urinary albumin; *HA* high level of urinary albumin

^a1^*p* < 0.05 vs. LP

^a2^*p* < 0.01 vs. LP

^b1^*p* < 0.05 vs. MP

^b2^*p* < 0.01 vs. MP by the Steel–Dwass test; bias, difference of the later measurement against the previous measurement

Urinary creatinine values were categorized into three groups: less than 50 mg/dl; 50 mg/dl or more but less than 90 mg/dl; and 90 mg/dl or more (Table 14). The two intraday comparisons and four interday comparisons all indicated considerable positive correlations in all groups.

**Table 14:** Intraday and interday correlation and variance of urinary creatinine of the same sample according to the mean urinary creatinine level

	Mean of 2 urinary creatinine			Regression line	*r*	*p*	Bias of uCr
	Range (mg/dl)	*n*	(mg/dl)				(mg/dl)
Intraday							
3–4 h							
LC1-2	< 50.0	30	35.8 (29.1–43.6)	2uCr = 1.001 × 1uCr-0.06834	0.99	< 0.0001	− 0.1− 0.2 to 0.1)
Mcl-2	50.0 to < 90.0	30	64.7(58.4-76.5)^a2^	2uCr = 0.9962 × 1uCr-0.2676	0.99	< 0.0001	− 0.1− 0.4 to 0.4)
HC1-2	≥ 90.0	28	128.1 (113.9–157.2)^a2b2^	2uCr = 1.013 × 1uCr-1.091	0.99	< 0.0001	0.5 (− 0.6 to 1.3)
3 min							
LC3-4	< 50.0	30	35.9 (28.7–43.7)	4uCr = 1.008 × 3uCr + 0.07271	0.99	< 0.0001	0.1 (− 0.1 to 0.4)
MC3-4	50.0 to < 90.0	29	64.4 (58.6–76.6)^a2^	4uCr = 0.9911 × 3uCr + 0.6046	0.99	< 0.0001	− 0.1(− 0.3 to 0.3)
HC3-4	≥ 90.0	29	126.9 (114.5–156.0)^a2b2^	4uCr = 0.9940 × 3uCr + 1.619	0.99	< 0.0001	0.5 (− 0.4 to 0.9)
Interday							
3–4 days							
LC1-3	< 50.0	30	35.3 (28.7–43.8)	3uCr = 0.9880 × 1uCr + 0.04098	0.99	< 0.0001	− 0.1 (− 0.2 to 0.1)
MC1-3	50.0 to < 90.0	29	64.5 (58.5–76.7)^a2^	3uCr = 1.023 × 1uCr-1.095	0.99	< 0.0001	0.3 (− 0.1− 0.6)^a1^
HC1-3	≥ 90.0	29	127.6 (113.6–154.1)^a2b2^	3uCr = 1.030 × 1uCr-3.276	0.99	< 0.0001	1.1 (− 0.5 to 2.3)
LC1-4	< 50.0	30	36.0 (29.0–43.8)	4uCr = 1.004 × 1uCr-0.1729	0.99	< 0.0001	0 (− 0.2 to 0.4)
MC1-4	50.0 to < 90.0	30	64.8 (58.6–76.7)^a2^	4uCr = 1.014 × 1uCr-0.4915	0.99	< 0.0001	0.3 (− 0.2 to 1.0)
HC1-4	≥ 90.0	28	128.0 (114.2–157.6)^a2b2^	4uCr = 1.023 × 1uCr-1.646	0.99	< 0.0001	1.1 (− 0.1 to 2.4)
3–4 days							
LC2-3	< 50.0	30	35.3 (28.8–43.6)	3uCr = 0.9859 × 2uCr + 0.1434	0.99	< 0.0001	0 (− 0.4 to 0.3)
MC2-3	50.0 to < 90.0	29	64.6 (58.3–76.4)^a2^	3uCr = 1.022 × 2uCr − 1.109	0.99	< 0.0001	0.5(0–0.7)^a2^
HC2-3	≥ 90.0	29	127.7 (114.0–154.6)^a2b2^	3uCr = 1.016 × 2uCr − 2.048	0.99	< 0.0001	0.6 (− 0.5 to 1.4)
LC2-4	< 50.0	30	35.9 (29.1–43.5)	4uCr = 1.002 × 2uCr − 0.09247	0.99	< 0.0001	0.1 (− 0.1 to 0.3)
MC2-4	50.0 to < 90.0	30	64.9 (58.5–76.5)^a2^	4uCr = 1.017 × 2uCr − 0.7118	0.99	< 0.0001	0.5 (0.2–0.8)^a2^
HC2-4	≥ 90.0	28	127.8 (115.0–158.3)^a2b2^	4uCr = 1.010 × 2uCr − 0.5403	0.99	< 0.0001	0.8 (− 0.3 to 1.7)

The numbers of 1, 2, 3, and 4 represent the first, second, third, and fourth measurements, respectively, of urinary creatinine. Data are presented as median (IQR)

*uCr* urinary creatinine, *LC* low level of urinary creatinine; *MC* medium level of urinary creatinine; *HC* high level of urinary creatinine

^a1^*p* < 0.05 vs. LP

^a2^*p* < 0.01 vs. LP

^b1^*p* < 0.05 vs. MP

^b2^*p* < 0.01 vs. MP by the Steel–Dwass test; bias, difference of the later measurement against the previous measurement

**Fig. 7:**
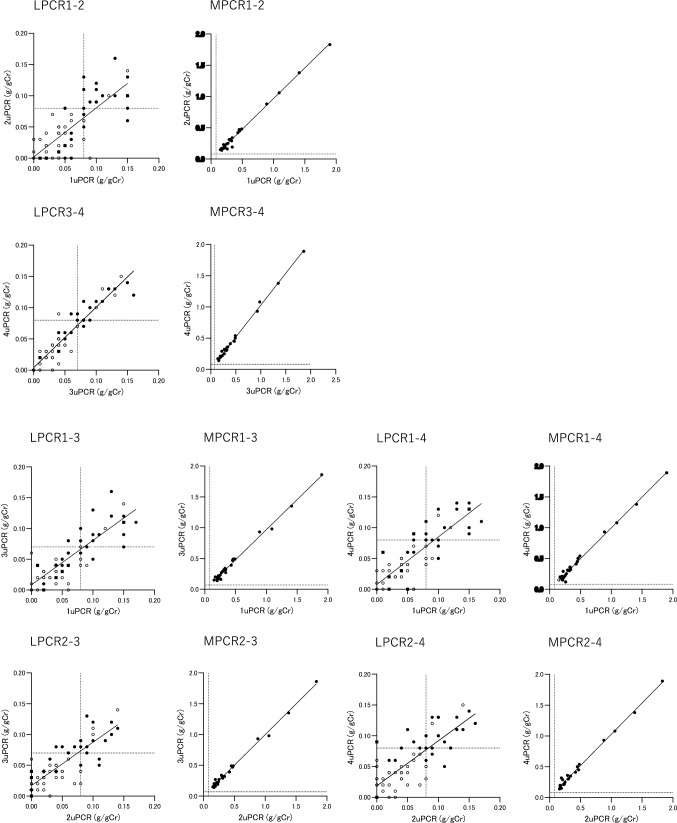
Intraday and interday correlation of urinary protein creatinine ratio of the same sample according to the mean of 2 uPCR levels. The symbols in the upper left of the figure correspond to the symbols in Table 15. ● Both of the two measurements were uACR 30 mg/gCr or more; ■ The previous measurement was uACR ≥ 30 mg/gCr, while the later measurement was uACR < 30 mg/gCr; ◆ The previous measurement was uACR < 30 mg/gCr, while the later measurement was uACR ≥ 30 mg/gCr; ○ both measurements were uACR < 30 mg/gCr. The vertical and horizontal dotted lines indicate the CO value of uPCR to determine uACR ≥ 30 mg/gCr for each measurement

We categorized the mean of uPCR values into two groups: less than 0.15 g/gCr and 0.15 g/gCr or more (Table 15, Fig. 7). For uPCR, the two intraday comparisons and four interday comparisons all indicated significant positive correlations in a both groups. For uACR, significant positive intraday and interday correlations were found in both groups.

**Table 15:** Intraday and interday correlation and variance of the uPCR and uACR of the same sample according to the mean uPCR values

						Mean of				Bias	Mean of	Mean of		*r*	*p*	Bias
		Mean of 2 uPCR			Mean of	2 urinary protein	Regression line			of uPCR	2 urinary albumin	2 uACR	Regression line			of uACR
		(g/gCr)	*n*	(g/gCr)	2 uCr/sCr	(mg/dl)		*r*	*p*	(g/gCr)	(mg/l)	(mg/gCr)				(mg/gCr)
Intraday																
3-4 h	LPCR1-2	< 0.15	63	0.05 (0.02–0.09)	58.7 (38.4–97.8)	3.1 (1.3–7.0)	2uPCR = 0.7859 × 1uPCR + 0.001936	0.78	< 0.0001	− 0.01 (− 0.03 to 0)	15.9 (7.1–30.8)	22.0 (12.0–39.3)	2uACR = 0.9843 × 1uACR − 1.229	0.98	< 0.0001	− 1.0 (− 2.0 to 0)
	MPCR1-2	≥ 0.15	25	0.30 (0.21–0.46)^**^	51.2 (36.1–85.1)	22.2 (10.0–29.8)**	2uPCR = 0.9818 × 1uPCR − 0.01493	0.99	< 0.0001	− 0.01 (− 0.03 to 0)	154.1 (44.3–200.0)**	192.0 (132.0–299.0)**	2uACR = 1.018 × 1uACR − 8.972	0.99	< 0.0001	− 2.0 (− 4.0 to 0)
3 min	LPCR3-4	< 0.15	65	0.05 (0.03–0.08)	57.2 (38.5–100.0)	3.0 (1.4–7.0)	4uPCR = 0.9669 × 3uPCR + 0.003974	0.94	< 0.0001	0(0–0.01)	14.9 (5.8–30.9)	21.0 (9.5–39.0)	4uACR = 1.002 × 3uACR − 0.6946	0.99	< 0.0001	0 (− 1.0 to 0)
	MPCR3-4	≥ 0.15	23	0.31 (0.21–0.48)^**^	51.9 (35.6–88.3)	23.3 (13.7–31.8)**	4uPCR = 1.022 × 3uPCR + 0.006523	0.99	< 0.0001	0.02 (− 0.01 to 0.03)	155.7 (72.7–199.2)**	197.0 (146.0–310.3)**	4uACR = 0.9974 × 3uACR + 0.8702	0.99	< 0.0001	0 (− 1.0 to 3.5)
Interday																
3–4 days	LPCR1-3	< 0.15	64	0.05 (0.03–0.08)	58.8 (38.5–97.3)	3.3 (1.4–7.2)	3uPCR = 0.7230 × 1uPCR + 0.008293	0.82	< 0.0001	− 0.01 (− 0.02 to 0)	15.5 (7.3–30.9)	23.1 (12.4–40.1)	3uACR = 0.9306 × 1uACR − 0.4581	0.96	< 0.0001	− 1.0 (− 3.3 to 1.0)
	MPCR1-3	≥ 0.15	24	0.31 (0.21–0.46)^**^	50.2 (35.8–87.7)	22.8 (12.1–31.4)**	3uPCR = 0.9773 × 1uPCR − 0.009794	0.99	< 0.0001	− 0.01 (− 0.04 to 0)	154.5 (52.3–207.2)**	193.8 (146.0–304.3)**	3uACR = 1.033 × 1uACR − 10.65	0.99	< 0.0001	− 0.5 (− 4.5 to 2.0)
	LPCR1-4	< 0.15	64	0.05 (0.03–0.09)	59.9 (38.3–100.0)	3.4 (1.5–7.3)	4uPCR = 0.7737 × 1uPCR + 0.007619	0.84	< 0.0001	0 (− 0.02 to 0.01)	15.1 (6.9–30.9)	21.6 (11.9–40.3)	4uACR = 0.9502 × 1uACR − 1.637	0.96	< 0.0001	− 1.0 (− 3.5 to 0)
	MPCR1-4	≥ 0.15	24	0.31 (0.21–0.46)^**^	50.2 (35.9–85.9)	22.9 (11.9–30.7)**	4uPCR = 1.002 × 1uPCR − 0.006477	0.99	< 0.0001	− 0.01 (− 0.02 to 0.02)	152.0 (44.9–198.8)**	194.0 (145.4–306.1)**	4uACR = 1.030 × 1uACR − 9.879	0.99	< 0.0001	− 1.0 (− 2.5 to 3.3)
3–4 days	LPCR2-3	< 0.15	63	0.05 (0.02–0.08)	58.6 (38.6–97.8)	3.1 (1.1–6.3)	3uPCR = 0.7046 × 2uPCR + 0.01701	0.81	< 0.0001	0 (− 0.01 to 0.02)	14.2 (6.7–28.3)	22.0 (10.8–38.5)	3uACR = 0.9276 × 2uACR + 1.318	0.94	< 0.0001	0 (− 0.5 to 2.0)
	MPCR2-3	≥ 0.15	25	0.30(0.19–0.47)^**^	51.6 (36.0–89.8)	21.7 (13.3–30.8)**	3uPCR = 0.9932 × 2uPCR + 0.005331	0.99	< 0.0001	0 (− 0.02 to 0.02)	154.3 (54.5–200.2)**	190.5 (132.5–297.0)**	3uACR = 1.015 × 2uACR − 1.886	0.99	< 0.0001	1.0 (− 3.0 to 5.0)
	LPCR2-4	< 0.15	65	0.05 (0.02–0.08)	58.7 (38.5–99.2)	2.9 (1.2–7.1)	4uPCR = 0.7315 × 2uPCR + 0.01863	0.80	< 0.0001	0 (− 0.01 to 0.02)	14.9 (6.7–30.1)	22.2 (11.0–40.0)	4uACR = 0.9762 × 2uACR − 0.5351	0.97	< 0.0001	1.0 (− 1.0 to 1.0)
	MPCR2-4	≥ 0.15	23	0.31 (0.23–0.47)^**^	51.6 (35.7–87.9)	21.9 (14.0–30.5)**	4uPCR = 1.016 × 2uPCR + 0.01199	0.99	< 0.0001	0 (− 0.01 to 0.05)	155.9 (72.1–198.1)**	193.5 (145.3–311.5)**	4uACR = 1.013 × 2uACR − 1.315	0.99	< 0.0001	3.0 (0–5.5)*

The numbers of 1, 2, 3, and 4 represent the first, second, third, and fourth measurements, respectively, of urinary protein, albumin, and creatinine. Data are presented as the median (IQR)

*uCr/sCr* urine-to-serum creatinine ratio; uACR, urinary albumin-to-creatinine ratio; *uPCR* urinary protein-to-creatinine ratio, *LPCR* low urinary albumin-to-creatinine ratio; *MPCR* medium urinary protein-to-creatinine ratio

*p < 0.01; **p < 0.001 vs. uPCR < 0.15 g/gCr by the Mann–Whitney's *U* test bias, difference of the later measurement against the previous measurement

The urinary protein concentration is useful to determine whether or not the uACR is ≥ 30 mg/gCr and the CO of urinary protein concentration was 3.3 to 4.0 mg/dl (Table 16). The CO value of uPCR to determine whether or not uACR is ≥ 30 mg/gCr was 0.08 g/gCr in the the first, second, and fourth measurements, and 0.07 g/gCr in the third measurement, each indicating a significantly higher ability to determine whether or not uACR is ≥ 30 mg/gCr, compared to the urinary protein concentration measured simultaneously. The CO value of uPCR to determine whether or not the mean of the four uACR measurements is ≥ 30 mg/gCr was 0.08 g/gCr in the first, second, and fourth measurements, and 0.07 g/gCr in the third measurement (Table 17). There was no significant difference in the values of ROC curve area between the four measurements.

**Table 16:** Cut-off value (YI) of the urinary protein and uPCR to discriminate the samples of uACR ≥ 30 mg/gCr

Test variables	1uP	2uP	3uP	4uP	1uPCR	2uPCR	3uPCR	4uPCR
uACR ≥ 30 mg/gCr	52	47	47	45	52	47	47	45
ROC curve area	0.767	0.800	0.821	0.860	0.888^a2^	0.895^a1^	0.920^a2^	0.948^a1^
Standard error	0.051	0.047	0.045	0.040	0.034	0.036	0.029	0.022
*p* value	< 0.0001	< 0.0001	< 0.0001	< 0.0001	< 0.0001	< 0.0001	< 0.0001	< 0.0001
CO(YI)^a^	4.0	3.3	3.4	3.6	0.08	0.08	0.07	0.08
Sensitivity	0.85	0.85	0.89	0.91	0.81	0.83	0.85	0.89
Specificity	0.64	0.68	0.68	0.72	0.81	0.85	0.85	0.88

The numbers of 1, 2, 3, and 4 represent the first, second, third, and fourth measurements, respectively, of urinary protein, albumin, and creatinine

*CO(YI)* cut-off value using Youden's index, *uP* urinary protein; *uACR* urinary albumin-to-creatinine ratio; *uPCR* urinary protein-to-creatinine ratio

^a1^*p* < 0.01 vs. urinary protein

^a2^*p* < 0.001 vs. urinary protein measured at the same time

^a^Unit of uP, mg/dl; Unit of uPCR, g/gCr

**Table 17:** Comparison of cut-off value (YI) of the uPCR to discriminate the samples of mean of 4 uACR ≥ 30 mg/gCr

Test variables	1uPCR	2uPCR	3uPCR	4uPCR
Mean of 4 uACR ≥ 30 mg/gCr	49	49	49	49
ROC curve area	0.927	0.891	0.918	0.929
Standard error	0.026	0.036	0.029	0.029
*p* value	< 0.0001	< 0.0001	< 0.0001	< 0.0001
CO(YI) (g/gCr)	0.08	0.08	0.07	0.08
Sensitivity	0.86	0.82	0.84	0.86
Specificity	0.82	0.87	0.87	0.92

The numbers of 1, 2, 3, and 4 represent the first, second, third, and fourth measurements, respectively, of urinary protein, albumin, and creatinine

*CO(YI)* cut-off value using Youden's index; *uACR* urinary albumin-to-creatinine ratio; *uPCR* urinary protein-to-creatinine ratio

For the group in which the mean value of the two measurements of uPCR was 0.15 g/gCr or more, both values were no less than the CO values, and in most cases, both of the two measurements resulted in a uACR ≥ 30 mg/gCr (Fig. 7). In the group of uPCR < 0.15 g/gCr, the median uACR was 21 to 23 mg/gCr, with the results indicating three different patterns: both measurements resulted in uACR ≥ 30 mg/gCr; only one measurement was uACR ≥ 30 mg/gCr; and both measurements were uACR < 30 mg/gCr. The median uPCR and uACR equivalent to a urinary protein concentration of less than 3 mg/gCr was 0.02 to 0.03 g/gCr and 13 to 15 mg/gCr, respectively. The uPCR mostly fluctuated in a range less than the CO value.

In this study, the CO of urinary protein concentration that determines whether or not uACR is ≥ 30 mg/gCr in non-diabetic diabetes (591 times) and diabetes (318 times) was 7 mg/dl (AUC 0.749 SE 0.020, sensitivity 0.67, specificity 0.73, *p* < 0.0001) and 6 mg/dl (AUC 0.781 SE 0.026, sensitivity 0.70, specificity 0.77, *p* < 0.0001), and the values of ROC curve area of urinary protein concentration were found to be significantly lower than those of uPCR (*p* < 0.0001), respectively.

## Discussion

This study examined whether the uPCR could differentiate normoalbuminuria and microalbuminuria in adults with lifestyle-related diseases. This study targeted cases with uPCR of < 0.50 g/gCr. The target was set because the uACR was significantly beyond 30 mg/gCr for cases in which the uPCR increased by > 0.50 g/gCr after the second measurement in this study, with a previous study reporting that there was a direct correlation between uACR and uPCR when uPCR was > 0.20 g/gCr [17]. Although it is predicted that the sensitivity of microalbuminuria detected based on proteinuria would increase with the inclusion of cases in which the uPCR is > 0.50 g/gCr, it is believed that the CO value would not change since the value with the greatest Youden’s index was selected as the CO value in this study. Moreover, it is believed that a CO of uPCR that can detect microalbuminuria with sufficient sensitivity for patients with little proteinuria is clinically necessary. It is also necessary to set a fixed value for the maximum limit of proteinuria when comparing the sensitivity to that in other groups.

Weaver et al.[19] examined the relationship between log (uACR) and log (uPCR), developed a formula for predicting the median uACR from the uPCR measured in the same day, and reported that the estimation was more accurate when the uPCR was more than 500 mg/gCr. The range for predicting uACR from uPCR becomes wider in the low ranges of proteinuria. This is partly because the uPCR is less accurate than the uACR in lower ranges [22]. In addition, uACR and uPCR have different values depending on the damaged site of the kidney and the diversity of the disease state is greater in the low urinary protein ranges in individual cases [19]. Although the ratio of the range of the first to third quartile for the median uACR in patients with low uPCR values was also wide, a relationship in which the median uACR increases as the uPCR increases was confirmed in the low ranges, with the slope of Δln (uACR)/Δln (uPCR) in the overall patients and in non-diabetic patients becoming steeper when the uPCR was ≥ 0.06 g/gCr. This time, we measured low levels of protein and examined whether it was possible to predict microalbuminuria.

The present study clarified that the uPCR was useful for differentiating uACR ≥ 30 mg/gCr or microalbuminuria, and that the CO of the uPCR is 0.09 g/gCr. According to the equations by Weaver et al. [19], the uPCR corresponding to a median ACR of 30 mg/gCr is 0.139 g/gCr, whereas the median ACR at uPCR 0.15 g/gCr was 35.5 mg/gCr (IQR16.0–65.8 mg/gCr). On the other hand, in the equation of this study, the uPCR corresponding to the median uACR of 30 mg/gCr was 0.077 g/gCr, whereas the median ACR corresponding to a uPCR of 0.15 g/gCr was 78 mg/gCr in the overall patients. The predicted median uACR in this study was higher than the predicted value determined by the equation of Weaver et al. at low uPCR levels, with a tendency for the gap to become smaller and then to eventually match when the uPCR increased. Further studies are needed to determine the cause of the gap; however, it is believed that this is the reason why the CO of the uPCR which can differentiate microalbuminuria from normoalbuminuria is lower than 0.15 g/gCr, which is supposed to be equivalent to uACR 30 mg/gCr according to the KDIGO guideline [1].

The ability to differentiate microalbuminuria based on uPCR significantly increased with the sum of two or three of uPCRs in comparison to a single uPCR. Therefore, the sum of two or three consecutive uPCRs was considered useful for differentiating microalbuminuria.

With respect to the factors affecting CO in differentiating microalbuminuria, it has been reported that a higher uACR/uPCR was associated with younger age [17–19], male sex [18, 19], non-white race [18], and diabetes mellitus [17–19]. This study indicated that age and sex did not affect the CO in non-diabetics. The difference in results may be attributed to the examination of the effects on the CO by dividing patients into non-diabetics and diabetics in this study.

In diabetics, the CO in males was lower than that in females and it is reported that males have a higher uACR/uPCR ratio than females, with sex being the most important modifier of the relationship between uACR and uPCR [19]. However, since there were few patients with diabetes in this study, further examination of the effect of sex is believed to be necessary.

With respect to the effect of the GFR category on CO in differentiating microalbuminuria, the CO tended to be higher due to the progression of the GFR category, which was 0.07 g/Cr in G1-3a and 0.10 g/g Cr in G3b-4 in non-diabetics. It has been reported that the uACR corresponding to a uPCR of 0.15 g/gCr is lower in G4 and G5 in comparison to eGFR > 30 ml/min/1.73m^2^ [19]. The reason for the increase in the CO may be attributed to a decrease in urinary protein selectivity, tubular proteinuria due to the progression of interstitial lesions, or the possible increase in urinary protein components, other than albumin, due to differences in protein reabsorption in the renal tubules [23]. On the other hand, it has also been reported that the ratio of albumin in the urinary protein increases as the GFR decreases [17, 18]. However, because the ratio of albumin in the urinary protein increases with urinary albumin levels of up to 500 mg/gCr and urinary protein levels of up to 1000 mg/gCr [17], the studies that included overt proteinuria may have been looking at the effect of urinary protein, which increased as the GFR category progressed.

As for the clinical application for uPCR to uACR conversion equations, Weaver et al. [19] recommend measuring the uACR when possible, and estimating the median as well as the 25th and 75th percentiles of the ACR, if it is not feasible. However, the measurement of the uACR in non-diabetic patients has not been given insurance coverage in Japan. By differentiating microalbuminuria with uPCR of 0.09 g/gCr in G1-4 or 0.07 g/gCr in G1-3a—rather than a uPCR of 0.15 g/gCr—although the specificity becomes lower, the sensitivity in detecting microalbuminuria increases. In particular, it becomes possible to detect microalbuminuria of > 100 mg/gCr, which is a relatively high level. This suggests that the clinical application of the prediction of the uACR from uPCR may potentially enable early intervention for patients with microalbuminuria.

On the other hand, when microalbuminuria was differentiated by uPCR of 0.09 or 0.07 g/gCr, false positive findings were detected more frequently in comparison to when microalbuminuria was differentiated by a uPCR of 0.15 g/gCr. However, false positive findings were more frequently observed in cases with a uACR of ≥ 10 mg/gCr in comparison to those with a uACR of < 10 mg/gCr. It is reported that the risk of cardiovascular disease and death increases at uACR levels of < 10 mg/gCr or albuminuria 10 mg/day [5, 6, 10], and a very low level of microalbuminuria is also a risk factor, independent of the kidney function, and the presence of hypertension and diabetes [6]. This suggests that the disadvantages of an increased false positive rate may be relatively low.

This study indicated the overall high degree of positive correlation between changes in the uPCR and uACR in patients with an initial uPCR < 0.15 or ≥ 0.15 g/gCr, suggesting that the uPCR may be useful for monitoring the course of microalbuminuria. This may be the reason why the ability to differentiate microalbuminuria is increased by the sum of multiple uPCR values in non-diabetic patients.

Among patients with non-diabetic lifestyle-related diseases, microalbuminuria was confirmed in 42.7% of cases that were negative proteinuria by dipstick analysis on the first measurement, 76.7% of the (±) cases, 81.0% of the (1+), and all (2+) cases. Normally, lifestyle improvement and health guidance are provided when proteinuria is (±) or higher at a checkup. In the event that proteinuria (±) persists, it is necessary to visit a medical institution. Since outpatients were targeted and the frequency of microalbuminuria was high despite proteinuria negativity in this study, it is believed that an early diagnosis and intervention for microalbuminuria in non-diabetic lifestyle-related diseases may be possible by determining the quantity of proteinuria, even it is only measured once while the patient is negative for proteinuria by dipstick analysis. While the sum of multiple measurements of urinary protein is useful for diagnosing microalbuminuria, it is desirable to make a tentative diagnosis after the first measurement to ensure that patients continue to visit the hospital. It is therefore necessary to evaluate the usefulness of multiple measurements including the cost–benefit.

The intra- and interday variance of uPCR and uACR of the same sample is influenced by urinary protein and urinary albumin concentrations, as the urinary creatinine has a considerable positive correlation and is nearly the same. In the group with urinary protein less than 3 mg/dl, the consecutive measurements indicated significant positive correlations. However, the measurements after several hours of refrigeration, several days of freezing, and thawing had insignificant or reduced correlations, indicating that specimen preservation may have influenced the measurement of traces of protein. In the group with a urinary protein concentration of 3 mg/dl or more, there was a significant positive interday and intraday correlation, which was reproducible. In the group with a low urinary albumin concentration of less than 15 mg/l, the median uACR was equivalent to that of normoalbuminuria, wherein the measurements had an intraday and interday positive correlation, which was relatively stable and reproducible.

The CO of uPCR to determine whether uACR is 30 mg/gCr or more was 0.07 to 0.08 g/gCr and underwent no intraday or interday changes. The reason for this is that when uPCR is > 0.15 g/gCr, in most cases, uACR is ≥ 30 mg/gCr, so the CO value mainly depends on the relation between the two values when uPCR is less than 0.15 g/gCr. When uPCR is less than 0.15 g/gCr, the median of urinary albumin was approximately 15 mg/l and uACR had a positive correlation. On the other hand, uPCR also had significant positive correlations as it includes cases with proteinuria of ≥ 3 mg/dl. When the urinary protein concentration is low, at less than 3 mg/dl, the correlation of uPCR is also expected to be poor; however, the median uPCR is low at 0.02 to 0.03 g/gCr, the median uACR corresponds to normoalbuminuria, and uPCR fluctuates within the range less than the CO to determine the microalbuminuria. Therefore, it is believed that the poor correlation of low urinary protein concentrations did not affect the CO value and discrimination ability of microalbuminuria.

In this study, the urinary protein was rapidly measured using morning urine and it was found that the CO of urinary protein concentration to determine whether uACR is 30 mg/gCr or more was 7 mg/dl for non-diabetes and 6 mg/dl for diabetes, respectively. Since they were 3 mg/dl or more, it is predicted that the CO value of uPCR to determine the microalbuminuria did not change even if measured the same day after refrigeration or after several days of cryopreservation. The CO of uPCR to discriminate microalbuminuria should be evaluated using uPCR because the urinary protein concentration depends on the urine dilution status. Although the urinary protein concentration should be considered in terms of the reproducibility of uPCR values in preserved specimens, the poor correlation of low urinary protein concentration has less impact on CO of uPCR in determining microalbuminuria with the sufficient urinary creatinine concentration, as the uPCR only fluctuates within the range below the CO.

The present study was associated with some limitations. Because the sample collection time and measurement/analysis method differed at each facility, the relationship between the uACR and uPCR may differ, making it necessary to set the CO of the uPCR for distinguishing microalbuminuria at each facility.

In conclusion, the uPCR may be useful for predicting microalbuminuria in non-diabetic adults with lifestyle-related diseases. In this study, the CO of the uPCR that predicted microalbuminuria in non-diabetic lifestyle-related diseases was 0.09 g/gCr for the GFR category of G1-4 and 0.07 g/gCr for the GFR category of G1-3a. It is anticipated that kidney failure and cardiovascular disease will be reduced by strengthening intervention for CKD due to non-diabetic lifestyle-related diseases from the early stages.
